# Climate Change-Related Temperature Impact on Human Health Risks of *Vibrio* Species in Bathing and Surface Water

**DOI:** 10.3390/microorganisms13081893

**Published:** 2025-08-14

**Authors:** Franciska M. Schets, Irene E. Pol-Hofstad, Harold H. J. L. van den Berg, Jack F. Schijven

**Affiliations:** Centre for Zoonoses and Environmental Microbiology, National Institute for Public Health and the Environment, P.O. Box 1, 3720 BA Bilthoven, The Netherlands; irene.pol@rivm.nl (I.E.P.-H.); harold.van.den.berg@rivm.nl (H.H.J.L.v.d.B.); jfschijven@gmail.com (J.F.S.)

**Keywords:** *Vibrio*, bathing water, surface water, probability of illness, global warming, risk assessment

## Abstract

*Vibrio* species are part of the indigenous microbial flora in marine, brackish and fresh water in moderate and tropical climates that thrive and multiply in water at elevated water temperatures. The number of human non-cholera *Vibrio* infections due to exposure to contaminated surface water increases worldwide. To study possible climate change-related changes in *Vibrio* concentrations, prevalent species, and risks of illness, water samples from coastal and inland water bodies in the Netherlands were tested in 2019–2021. Data were combined with data from previous studies in 2009–2012 in order to develop a regression model to predict current and future risks of *Vibrio* illness. Year-to-year and site-specific variations in *Vibrio* concentrations and water temperature were observed, but there was no trend of increasing *Vibrio* concentrations or water temperature over time. In 2019–2021, *Vibrio* species distribution had not changed since 2009–2012; *V. alginolyticus* and *V. parahaemolyticus* were still the dominant species. Statistical analysis demonstrated a significant effect of water temperature on *Vibrio* concentrations. The model predicted a concentration increase of a factor of 1.5 for each degree Celsius temperature increase. Predicted risks of illness were higher at higher water temperatures, and higher for children than for adults. Based on the most recent climate change scenarios for the Netherlands, the risks of *Vibrio* illness will increase with factors ranging from 1.6 to 7.6 in 2050 and 2100. These outcomes warrant adequate information about *Vibrio* risks to water managers, public health workers and the general public.

## 1. Introduction

*Vibrio* species are halophilic bacteria that are part of the indigenous aquatic microbial flora in marine, brackish and fresh water in both moderate and tropical climates. *Vibrio* species have previously been isolated from water, sediment, plankton and shellfish [1,2]. Presence and growth of *Vibrio* in water depend on various factors, including which *Vibrio* species, but water temperature and salinity are considered the most important [1,3]. Most *Vibrio* species thrive and multiply in water at elevated water temperatures, generally above 15 °C [4], with optimum values ranging from 20 to 36 °C [4,5,6]. *Vibrio* species tolerate a range of salinities, with species-dependent optimum values and preferences. For instance, *V. vulnificus* and *V. parahaemolyticus* prefer salinity below 14–20 g/L [4], whereas *V. alginolyticus* prefers 20–40 g/L [6], and *V. cholerae* has an optimum growth at 20–25 g/L, but is also able to grow in freshwater, in contrast to other *Vibrio* species [7,8].

About 12 *Vibrio* species are associated with human illness, including *V. cholerae*, *V. parahaemolyticus*, *V. alginolyticus*, *V. vulnificus*, *V. damsela*, *V. hollisae*, *V. mimicus*, *V. metshnikovii* and *V. fluvialis* [9]. Human infections with *Vibrio* may occur after exposure to, or ingestion of, contaminated surface water or bathing water, consumption of raw or undercooked food (particularly shellfish), or during the handling of fish, crustaceans and shellfish, if wounds are present or occur. In general, exposure to contaminated surface water and bathing water may result in ear and wound infections, whereas consumption of contaminated seafood may lead to gastroenteritis, and handling of contaminated seafood can result in wound infections [10,11].

The number of human non-cholera *Vibrio* infections has increased worldwide [1,2,12,13,14], and since 2006, this increase has also been observed in Europe, where it is particularly related to exposure to surface water [15,16,17,18,19]. Worldwide, the consumption of crustaceans and shellfish has increased, which is accompanied by concerns about *Vibrio*-related food safety [17,20].

A clear relationship between *Vibrio* concentrations and water temperature has been demonstrated in various studies, with higher concentrations at higher temperatures [12,21,22,23]. Due to this relationship, it is likely that climate change, which leads to higher average air and water temperatures, causes increased occurrence and concentrations of *Vibrio* in surface water, which may lead to an increased risk of *Vibrio* infections [13,14,17,24,25,26]. Increased water temperatures in moderate climate regions, such as Northwest Europe, may also change conditions into those favourable for *Vibrio* species that were previously rare and of limited public health concern, such as *V. vulnificus*. This species causes serious infections with a high mortality rate [27] and is particularly of concern in subtropical regions such as South-East USA, while it is currently displaying a geographical shift towards higher latitudes [14].

The presence of *Vibrio* species in surface water in the Netherlands has been demonstrated in 2009–2012, with *V. alginolyticus* and *V. parahaemolyticus* as the most frequently detected species [28,29]. Other recent studies have demonstrated the presence of *Vibrio* species in surface water in several other European countries, such as Germany [30], Belgium [31], Italy [32], and Serbia [33].

Based on the data from the Dutch studies [28], an empirical equation was derived for predicting *Vibrio* concentrations as a function of temperature, salinity and acidity of the water. Based on the climate scenarios from the Royal Dutch Meteorological Institute (KNMI) [34], elevated *Vibrio* concentrations were predicted, as well as an increased risk of gastrointestinal illness due to *V. parahaemolyticus* infections after exposure to marine, brackish and fresh surface water [29].

To study possible climate change-related changes in *Vibrio* concentrations and prevalent species in the Netherlands, and thus in the associated risks of humans contracting *Vibrio* infections through exposure to (marine) surface water, additional data were collected during 2019–2021. Data were used to create a prediction model and assess risks of *Vibrio* infections related to the latest climate change scenarios published by KNMI in 2023 [35].

## 2. Materials and Methods

### 2.1. Sampling Sites

Water samples were taken at official bathing sites in three waterbodies in the Netherlands: Eastern Scheldt (Oesterdam Westzijde badstrand; latitude 51.4782, longitude 4.2193), Wadden Sea (Harlingenstrand; latitude 53.1669, longitude 5.4160) and North Sea (Bergen aan Zee; latitude 52.6641, longitude 4.6276). Open sea samples were taken further away from the coast, from the Eastern Scheldt (longitude 51.4911, latitude 4.1951), and the Wadden Sea (longitude 53.3461, latitude 5.4421). Open sea samples were not taken from the North Sea for logistical reasons. However, open sea samples were taken from Veerse Meer (Bastiaan de Langeplaat; longitude 51.5254, latitude 3.7145), which is very close to one of the official bathing sites in this waterbody (De Piet badstrand; latitude 51.5301, longitude 3.7268), and samples thus represent both open water and bathing water.

### 2.2. Sampling

Samples (1 litre volumes) were taken according to ISO 19458:2006 [36]. At official bathing sites, water samples were taken at the beach, and open sea water samples were taken from ships. Water temperature, acidity and conductivity were measured on site at the time of sampling. For open sea water samples, measurements of acidity were not performed in 2019, whereas measurements of conductivity were not performed in 2019 and 2020 for logistical reasons. In 2019, samples were taken during May–October, and in 2020 and 2021, samples were taken from May through December. Samples were taken at weekly (July–September), biweekly (May–June, October) or monthly intervals (November–December). All samples were cooled in refrigerators (3–8 °C) immediately after sampling until cooled transport to the laboratory, where they were stored at 3–8 °C until analyses, which started within 24–36 h after sampling.

### 2.3. Analyses

Samples were analysed according to an in-house procedure that was based on ISO 21872-1:2017 [37]. Briefly: samples were enriched by incubating three or five subsequent volumes or dilutions of the samples in Alkaline Saline Peptone Water (ASPW; according to ISO 21872-1:2017 [37]) for 18 ± 1 h at 36 ± 2 °C. Tested sample volumes were initially 50, 10, 1, 0.1, and 0.01 mL, but were adjusted during the seasons as water temperatures and *Vibrio* concentrations increased and decreased again. After incubation, enriched cultures were plated onto Thiosulphate Citrate Bile Sucrose Agar (TCBS; Oxoid CM0333b, Thermo Fisher Scientific, Nieuwegein, The Netherlands) and incubated for 24 ± 3 h at 36 ± 2 °C.

Per sample, all positive volumes or dilutions that displayed colonies characteristic of *Vibrio* on TCBS were confirmed by growing pure cultures of a maximum of five green (e.g., *V. parahaemolyticus*, *V. vulnificus*) and five yellow (e.g., *V. mimicus*, *V. fluvialis*, *V. cholerae*) colonies on Saline Nutrient Agar (SNA; according to ISO 21872-1:2017 [37]), which was incubated for 24 ± 3 h at 36 ± 2 °C. At least one green and/or yellow colony per positive volume or dilution was isolated for confirmation. Subsequently, the pure cultures were identified by using an API20E identification test strip (bioMerieux Benelux B.V., Amersfoort, The Netherlands, no. 20100) and an oxidase test (BD BBL DrySlide, no. 231746). If isolates were identified as either *V. cholerae, V. parahaemolyticus*, or *V. vulnificus*, further confirmation was performed by PCR testing for specific genes; *ctxA* and *toxR* for *V. cholerae* [28], *gyrB for V. parahaemolyticus* [38], and *vvh* for *V. vulnificus* (primers [39], probe in-house).

### 2.4. Statistical Analyses

Based on the *Vibrio* concentrations measured in bathing water and at open sea in 2019–2021, and *Vibrio* concentrations measured in bathing water in previous studies [28,29], a statistical model was developed to predict *Vibrio* concentrations for site–water type–year combinations.

Firstly, the 2019–2021 data were used to perform maximum likelihood estimations using Mathematica (v. 13.0.1.0, Wolfram Inc., Champaign, IL, USA) in order to obtain the best estimates of the *Vibrio* concentrations in water. Next, a linear mixed effect analysis was conducted in R (version 4.1.2 (1 November 2021), R Foundation for Statistical Computing, Vienna, Austria. URL https://www.R-project.org/, accessed on 18 February 2025) for all log transformed bathing water concentrations from 2009 to 2012 and from 2019 to 2021 combined (Bird Hippy and lmerTest) [40], with temperature as a fixed effect. The combination of site, water type, and year was included as a random effect. Splitting site, water type and year into separate variables would have divided the data into too small sets of data to obtain meaningful effects.

The applied model equation for the prediction of *Vibrio* concentrations was:

^10^log(*MPN*) = *a*_0_ + *a*_1_ × *T* + *ε*_0_ + *ε*_1_ (1)

In which *MPN* is the *Vibrio* concentration (MPN/L), *T* the water temperature (°C), *a*_0_ and *a*_1_ are coefficients for background *Vibrio* concentrations and effect of temperature, respectively, *ε*_0_ is the random effect of the site–water type–year combination, and *ε*_1_ is the residual random effect. Coefficients *a*_0_, *a*_1_, *ε*_0_ and *ε*_1_ are normally distributed (Table 1).

Equation (1) was used to predict *Vibrio* concentrations for various water temperature scenarios, including 10 °C, 18 °C, 20 °C, 22 °C and 25 °C. The range of 18–25 °C reflects the water temperature in Dutch coastal waters during the months in which swimming is common. The temperature of 10 °C, which is approximately the annual average, was included as a low temperature at which no growth of *Vibrio* is expected [4].

### 2.5. Quantitative Microbial Risk Assessments (QMRA)

QMRA was conducted using the estimated total *Vibrio* concentrations in the mentioned temperature scenarios and for one single swimming event, in order to assess the risk of gastrointestinal illness due to swallowing water contaminated with *Vibrio*. The dose-response relation for *V. parahaemolyticus* illness [41,42,43] was used since dose-response relations for other relevant *Vibrio* species and other transmission routes, such as exposure of skin and ears, are unknown. The gamma-distributed data of the amount of water ingested per swimming event for children (*V_child_*), women (*V_woman_*) and men (*V_man_*) obtained by Schets et al. [44] were used (Table 1). The equation for dose (*D*), number of ingested *Vibrio* is:

*D* = *C* × *V* (2)

In which *C* is the *Vibrio* concentration in water (number per litre), and *V* is the ingested volume of water (litre) per swimming event for a child, woman or man. Risk of illness (*P*) was calculated as [41]:

*P* = 1 − (1 + *D*/*β)^α^* (3)

See Table 1 for values and sources of parameters alpha (*α*) and beta (*β*).

### 2.6. Climate Change Scenarios

In 2023, KNMI published four new climate scenarios for the Netherlands [35], which replaced the 2014 scenarios [34]. These new scenarios consider high and low carbon dioxide emissions in combination with a drier or a wetter climate. The low emission wet (Ln) scenarios predict a mean temperature increase of +1.1 °C in 2050 as well as in 2100, and the low emission dry (Ld) scenarios predict a mean temperature increase of +1.2 °C in both 2050 and 2100. The predicted mean temperature increase is +1.7 °C in 2050 and +4.7 °C in 2100 according to the high emission wet scenario (Hn), and +2.1 °C in 2050 and +5.1 °C in 2100 according to the high emission dry scenario (Hd). Assuming that water temperature rises with air temperature, it can be deduced that, consequently, there will be more days per year on which swimming is possible, as a result of climate change.

## 3. Results

### 3.1. Vibrio Concentrations and Water Temperature

**Figure 1:**
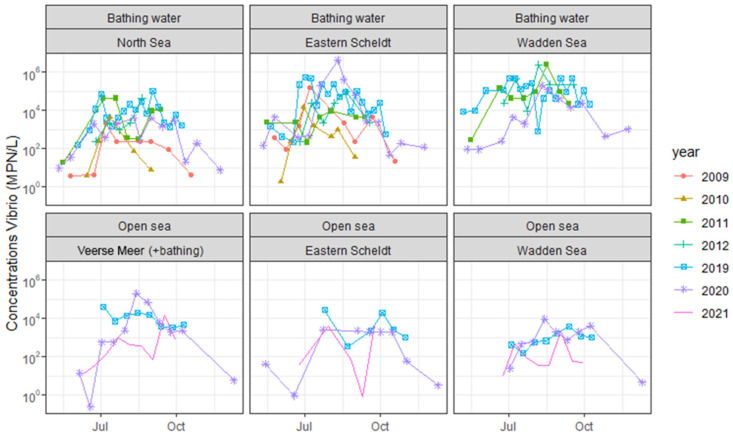
*Vibrio* concentrations at bathing sites and open sea in The Netherlands, 2009–2021.

**Figure 2:**
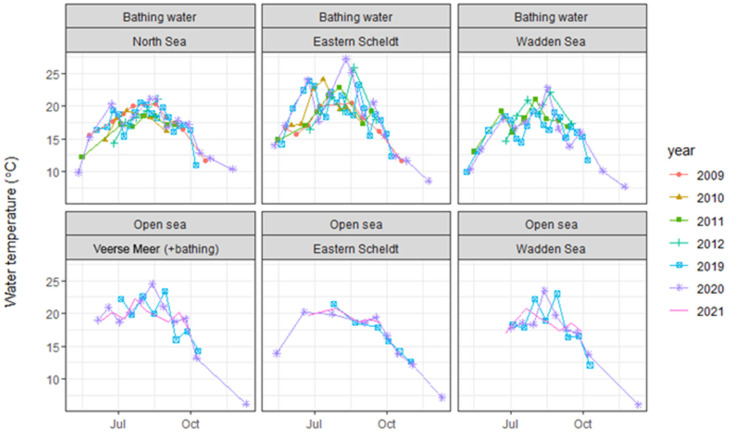
Water temperature at bathing sites and open sea in the Netherlands, 2009–2021.

*Vibrio* species were present in 163 of the 180 water samples (91%) examined during the 2019–2021 study (93/97 bathing water (96%), 70/83 open sea (84%)). Seasonality was observed for the *Vibrio* concentration and water temperature at all sites, with low values in spring, high values in summer and declining values in the late summer and autumn. This holds for both the data from the current study and the historical data from 2009 to 2012 (Figure 1 and Figure 2). At official bathing sites, *Vibrio* concentrations were generally higher in the Eastern Scheldt and the Wadden Sea than in the North Sea. Concentrations were generally lower at open sea than at bathing sites. At Veerse Meer, concentrations were in the same order of magnitude as those at the official bathing sites in Eastern Scheldt and Wadden Sea (Table 2).

Time series of *Vibrio* concentrations in both bathing water and open sea showed that there were differences between sites, water types and years, but there were no obvious trends (Figure 1). The time series of water temperatures (Figure 2) and *Vibrio* concentrations (Figure 1) showed strong similarities and suggested an increase in the *Vibrio* concentration when the water temperature increased. The statistical analysis confirmed a significant effect of water temperature on the *Vibrio* concentration, resulting in increased *Vibrio* concentrations at increased water temperatures. The residual variance *ε*_1_ and the variance attributable to the site–water type–year combination *ε*_0_ were about equal (Table 1).

### 3.2. Vibrio Species

In 2019–2021, a total of 1268 isolates were obtained, of which 667 were isolated from the bathing water samples. Of the bathing water isolates, 492 belonged to the *Vibrio* genus. The most frequently isolated species was *V. alginolyticus* (77%), followed by *V. parahaemolyticus* (19%). *V. vulnificus*, *V. fluvialis*, *V. cholerae* non-O1/non-O139, and *V. mimicus* were isolated occasionally. From the open sea samples, 601 isolates were obtained, of which 353 belonged to the *Vibrio* genus. The most frequently isolated species was again *V. alginolyticus* (72%), followed by *V. parahaemolyticus* (24%). *V. vulnificus* and *V. fluvialis* were isolated occasionally. The species distribution varied per site, with *V. alginolyticus* being the dominant species in the North Sea and the Eastern Scheldt, whereas in the Wadden Sea, the fraction of *V. parahaemolyticus* was larger, although it was not the dominant species in all the years studied. In Veerse Meer, *V. alginolyticus* and *V. parahaemolyticus* were both equally dominant (Table 3). Comparison of the 2019–2021 bathing water isolates with the 2009–2012 bathing water isolates [28,29] showed that the overall species distribution had not changed, although the fraction of *V. cholerae* non-O1/non-O139 had slightly decreased. Other minor differences reflect year-to-year variation.

### 3.3. Vibrio Concentrations and Risk of Illness in Different Scenarios

**Figure 3:**
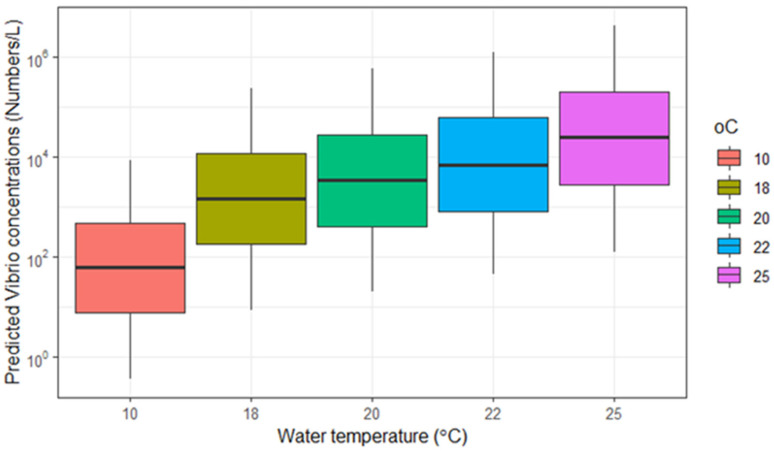
Predicted *Vibrio* concentrations in water (number/L) in water temperature scenarios of 10 °C, 18 °C, 20 °C, 22 °C and 25 °C for site–water type–year combinations. The boxplots display the median (horizontal line), quartiles (boxes), and the 5–95 percentiles (whiskers).

Using Equation (1) to predict the *Vibrio* concentration in water of 10 °C, 18 °C, 20 °C, 22 °C and 25 °C for a random site–water type–year combination, showed that *Vibrio* concentrations increased with a factor 10^0.017^, which equals a factor 1.5, for each degree Celcius temperature increase (Figure 3).

#### 3.3.1. Water Temperature Scenarios

**Figure 4:**
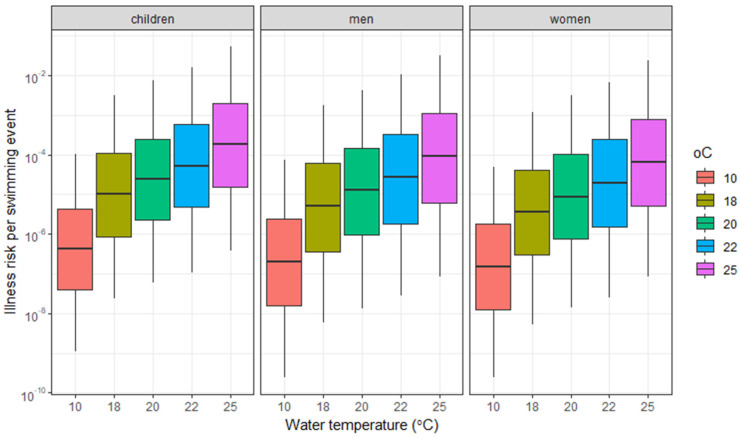
Estimated risk of *Vibrio* illness for water ingestion during one swimming event for *V. parahaemolyticus* only, for children, men and women, in different water temperature scenarios.

Risks of illness resulting from water ingestion while swimming in surface water contaminated with *Vibrio* were estimated using the parameters shown in Table 1, and the predicted *Vibrio* concentrations, which were calculated with Equation (1) and shown in Table 4. The 5–95 percentile values in this table reflect the range of predicted concentrations. Table 5 and Figure 4 show the risk of illness for children, women and men per single swimming event in different water temperature scenarios. The increased risk of increasing water temperature is clearly visible. In agreement with the level of exposure (i.e., the amount of water ingested), the risks are highest for children, followed by men and women. At a water temperature of 10 °C the 95-percentile values of the risks range from 5.1 × 10^−5^ to 1.1 × 10^−4^, at 18 °C, they range from 1.3 × 10^−3^ to 2.9 × 10^−3^, and at 25 °C from 2.5 × 10^−2^ to 5.0 × 10^−2^, showing an increased risk at higher water temperatures.

#### 3.3.2. Climate Change Scenarios

Based on the assumption that water temperature rises with air temperature, and the predicted increase in *Vibrio* concentrations with a factor of 1.5 for each degree Celsius temperature increase, the associated risk of illness would increase with a maximum factor of 1.5 per degree Celsius temperature as long as the risk is less than about 10%. At higher risks, the temperature effect is less because the dose response curve asymptotically approaches 1, hence the designation of “maximum” increase of the risk with temperature. For the latest climate scenarios for The Netherlands [33] the risks would increase with a maximum factor of 1.6 (Ln, 2050, 2100), 1.8 (Ld, 2050, 2100), 2.6 (Hn, 2050), 3.2 (Hd, 2050), 7.0 (Hn, 2100), and 7.6 (Hd, 2100).

## 4. Discussion

*Vibrio* concentrations in the studied bathing waters and the connected open sea varied from year to year and per site. There was no trend of increasing concentrations over time from 2009 to 2021. Measured water temperatures did not show an increasing trend either; however, the considered time frame (2009–2021) was probably too short to be able to detect such an increase. Other reports that do mention an increase in water temperature have used much larger datasets that comprise a much longer time period. For example, Brehm et al. [17] observed an increase in the water temperature in the Baltic Sea of 0.56 °C from 1982 to 2019. They, however, used large numbers of satellite data instead of incidentally measured water temperatures by samplers, which were performed in the current study. Reported observations of water temperature increase in the Netherlands were also based on large long-term datasets. For instance, from 1910 to 2019, the water temperature in the rivers Meuse and Rhine increased by 2.4 °C and 2.9 °C, respectively (Temperatuur oppervlaktewater, 1910–2019|Compendium voor de Leefomgeving, https://www.clo.nl/indicatoren/nl056605-temperatuur-oppervlaktewater-1910-2019) (Environmental Data Compendium, this page is only in Dutch, accessed on 18 February 2025). The observed average increase of the water temperature in the Dutch North Sea of 0.5 °C is also based on long-term measurements, from 1870 to 2022 (Decadal average sea surface temperature anomaly in different European seas (1870 to 2022)—European Environment Agency (europa.eu), https://www.eea.europa.eu/en/analysis/maps-and-charts/decadal-average-sea-surface-temperature-5, accessed on 29 July 2025).

Statistical analysis showed that all *Vibrio* concentrations in the studied water bodies in 2009–2021 were highly temperature dependent. The model for the *Vibrio* concentration demonstrated a linear relation between water temperature and *Vibrio* concentrations, in agreement with other studies that showed that temperature is the main driver of *Vibrio* abundance [4,14,19,45,46]. Although salinity, which is directly related to conductivity, and acidity are also factors that influence *Vibrio* growth [3,13,19,47], the data obtained in this study were too limited for inclusion in the statistical analysis.

Even though changes in precipitation patterns due to climate change may have an effect on *Vibrio* abundance through their effect on salinity and nutrient availability [13,14,22], they were not included in this study. Historical precipitation data in the Netherlands, from the 1900s on, show an increase in the mean summer precipitation [35]. The KNMI 2023 climate change scenarios predict a decrease in summer precipitation in 2050, with the largest effect in the Hn scenario and almost no change in the Ln scenario, becoming a trend around 2040. There is a first indication that the summer precipitation increase is already slowing down [35]. Thus far, changes in precipitation patterns were too limited to study any effect during the relatively short time frame of this study.

Higher *Vibrio* concentrations in water resulted in higher predicted risks upon exposure to this water. For risks below approximately 0.1, the effect of water temperature on the risk was the same as the effect of water temperature on the *Vibrio* concentration. For risks higher than 0.1, the effect of water temperature was less due to the non-linear relationship between concentration and risk. Based on the amount of water that is ingested during swimming [44], the dose-response relation for *V. parahaemolyticus* [41,42,43], and the predicted *Vibrio* concentrations, an estimated 1–3 persons per 1000 persons would become ill per swimming event at a water temperature of 18 °C. This number would increase to 3–5 persons per 100 persons per swimming event at a water temperature of 25 °C (95-percentile values). Since the dose response relation for *V. parahaemolyticus* and water ingestion was used in the risk assessments, the assessed risks concern gastrointestinal conditions. These risks are relatively high, especially when compared to the tolerated risk of infection (not illness) for drinking water consumption in the Netherlands [48]. However, the estimated *Vibrio* concentrations used in the risk assessment comprised the total *Vibrio* population, including other species than *V. parahaemolyticus*. Consequently, the risk of gastrointestinal illness is probably overestimated, although most other *Vibrio* species that were detected are also capable of causing gastroenteritis [1]. This includes *V. alginolyticus,* the most frequently detected species, although literature reports on such infections are limited [49,50,51,52].

*Vibrio* infections other than cholera are not notifiable in the Netherlands, and there is no active surveillance for such infections. However, other surveillance and registration systems occasionally included human *Vibrio* infections, of which *V. alginolyticus* and *V. parahaemolyticus* were the major aetiology, most frequently isolated from infected wounds [53]. These observations suggest that the calculated risks are indeed an overestimation. The risk calculations do, however, show that the risk increases with increasing water temperature, which is a relevant observation related to climate change-induced temperature increase.

Other *Vibrio* species and other exposure routes, resulting, for instance, in wound and ear infections, are at least as relevant with respect to swimming in surface water, although the lack of dose-response relations makes it impossible to calculate the risk of illness or infection for these *Vibrio*–transmission route combinations. However, an increased risk of wound and ear infections is to be expected, based on the expected increased *Vibrio* concentrations in water as a result of increased water temperatures due to climate change.

In bathing water and open sea, *V. alginolyticus* was the most prevalent species, followed by *V. parahaemolyticus*. The occurrence of these *Vibrio* species was constant per site and over time, although minor year-to-year variations were observed. These observations were in concordance with observations from previous studies in the Netherlands and reflect their ability to tolerate salinity levels that are common in Dutch coastal waters [28,29]. Throughout the years, other *Vibrio* species were only occasionally detected. A shift towards more virulent species, such as *V. vulnificus*, was not observed. *V. vulnificus* causes serious infections, particularly in areas with higher water temperatures [25]. An increase in the prevalence of *V. vulnificus* in the studied waterbodies in the Netherlands and an expansion of the geographical range of this species may not have occurred because the water temperature is not high enough, and salinity is generally higher than required for optimal growth of *V. vulnificus*. Moreover, optimal growth of *V. vulnificus* seems to require a complex, and often site-specific, combination of water temperature and salinity [54], which may not exist in the Netherlands thus far.

The KNMI 2023 climate scenarios [35] do not only predict an increase in temperature, but also an increase in the number of summer days (maximum temperature ≥ 25 °C) and tropical days (maximum temperature ≥ 30 °C), which is likely to result in more swimming activities and a prolonged bathing season, during which persons may be exposed to *Vibrio*. Since the *Vibrio* concentrations in water are expected to increase, the overall risks of contracting a *Vibrio* infection through ingestion of contaminated water while swimming in surface water in the Netherlands are also expected to increase over time. The results of the model used in this study are in line with the predictions made by Trinanes & Martinez-Urtaza [55]. They used models to create scenarios for the spatial and temporal distribution of non-cholera *Vibrio* species worldwide. They used climate, population and socio-economic data in their models. In the worst-case scenario, with projections for 2015–2100, an increase of 38,000 km coastline with favourable circumstances for *Vibrio* growth was predicted, as well as a one-month longer season during which *Vibrio* infections are to be expected. In Europe, the highest rate of expansion of *Vibrio* was predicted for the northern part of Europe, particularly the Baltic Sea area. According to their scenarios, the population at risk of contracting a *Vibrio* infection increased between 1980 and 2020, after which the growth trend is expected to weaken to a more moderate rise. Additionally, the models predict that the morbidity due to *Vibrio* will be more or less stable in the coming decades.

## 5. Conclusions

From 2009 to 2021, neither a trend of increasing *Vibrio* concentrations nor a change in the abundant *Vibrio* species in bathing water and open sea was observed in the Netherlands, implying that the risk of *Vibrio* illness did not increase during this period. However, based on the results from the prediction model for *Vibrio* concentrations in surface water in the Netherlands, and the latest climate change scenarios for the Netherlands, increasing *Vibrio* concentrations and risks of illness are to be expected in 2050 and 2100. These KNMI 2023 climate scenarios are based on the IPCC Sixth Assessment Report [56] and may therefore apply to a larger geographical area in Northern Europe, thus extending the value of our predictions beyond the Netherlands. Increasing *Vibrio* risks warrant that water managers, public health professionals and the general public are adequately informed about the risks resulting from exposure to surface water, taking into account different infection routes.

## Figures and Tables

**Table 1:** Parameter values for *Vibrio* risk assessment in water.

Model Parameter	Dimension	Value	Mean	Median	5–95%	References
variable						
*a*_0_	^10^log(MPN/L)	N ^2^ (0.021, 0.36)				Equation (1)
*a*_1_	^10^log(MPN/L)/°C	N (0.17, 0.017)				Equation (1)
*ε*_0_	^10^log(MPN/L)	0.89				Equation (1)
*ε*_1_	^10^log(MPN/L)	0.90				Equation (1)
dose response ^1^						
*α*		−0.6				[40,41]
*β*		1.3 × 10^6^				[40,41]
water ingestion						
*V_child_*	mL	G ^3^ (0.64, 58)	38	20	0.51–133	[42]
*V_woman_*	mL	G (0.45, 60)	18	8.1	0.076–67	[42]
*V_man_*	mL	G (0.51, 35)	27	11	0.062–110	[42]

^1^ Based on dose-response parameters for *V. parahaemolyticus*. ^2^ Normal distribution. ^3^ Gamma distribution.

**Table 2:** *Vibrio* concentrations and physical-chemical parameters in bathing water and open sea in the Netherlands.

	Site	North Sea—Bathing Site		Eastern Scheldt—Bathing Site		Eastern Scheldt—Open Sea			Wadden Sea—Bathing Site		Wadden Sea—Open Sea			Veerse Meer—Bathing Site/Open Sea		
Parameter	Year	2019	2020	2019	2020	2019	2020	2021	2019	2020	2019	2020	2021	2019	2020	2021
no. samples		20	13	19	13	6	10	7	19	13	8	11	10	8	12	11
*Vibrio* concentration (MPN/L)	min	0	9.2	2.3 × 10^2^	4.7 × 10^1^	1.5 × 10^2^	3.2	0	5.8 × 10^2^	0	5.5 × 10^1^	0	0	1.2 × 10^3^	0	0
	max	8.4 × 10^4^	3.9 × 10^4^	4.2 × 10^5^	4.3 × 10^6^	2.2 × 10^4^	1.2 × 10^4^	1.5 × 10^4^	4.6 × 10^5^	1.5 × 10^6^	1.2 × 10^3^	9.2 × 10^3^	4.3 × 10^3^	1.4 × 10^4^	2.0 × 10^5^	1.4 × 10^4^
	average	9.6 × 10^3^	5.3 × 10^3^	6.6 × 10^4^	3.9 × 10^5^	4.9 × 10^3^	2.5 × 10^3^	3.4 × 10^3^	1.3 × 10^5^	2.8 × 10^5^	4.2 × 10^2^	1.9 × 10^3^	7.8 × 10^2^	4.7 × 10^3^	2.5 × 10^5^	2.3 × 10^3^
	median	2.8 × 10^3^	1.9 × 10^3^	2.8 × 10^3^	4.2 × 10^3^	1.5 × 10^3^	2.0 × 10^3^	2.2 × 10^2^	4.5 × 10^4^	3.2 × 10^4^	3.0 × 10^2^	6.6 × 10^2^	1.5 × 10^2^	3.6 × 10^3^	1.9 × 10^3^	3.9 × 10^2^
Water temperature (°C)	min	11.0	9.9	12.4	8.6	12.6	7.2	7.2	10.0	6.9	12.1	6.1	7.3	14.3	6.2	7.2
	max	20.6	21.1	23.8	27.2	21.4	24.2	20.7	19.2	22.8	23.0	23.5	20.8	23.4	24.5	22.2
	average	17.1	16.0	19.3	18.0	16.8	17.0	16.8	16.1	15.0	18.2	17.0	16.9	19.4	19.0	18.0
	median	17.0	17.0	19.6	17.7	16.8	17.6	18.7	16.4	16.0	18.0	17.8	18.0	19.8	19.1	19.1
acidity (pH)	min	7.9	8.0	8.0	8.0	-	5.6	7.2	6.8	7.5	-	5.1	6.4	-	7.3	6.6
	max	8.3	8.2	8.6	8.4	-	8.3	8.4	8.0	8.0	-	8.3	8.4	-	8.6	8.6
	average	8.2	8.0	8.3	8.0	-	8.0	7.9	7.8	8.0	-	8.0	7.6	-	8.0	7.8
	median	8.2	8.1	8.3	8.2	-	8.0	8.1	7.8	7.9	-	8.1	7.6	-	8.3	8.2
conductivity (mS/cm)	min	42.4	40.7	-	42.6	-	-	47.7	28.9	17.2	-	-	47.6	-	-	43.4
	max	51.0	43.6	50.0	46.5	-	-	56.9	47.2	41.8	-	-	59.2	-	-	56.3
	average	49.0	43.0	50.0	45.0	-	-	52.6	37.2	31.0	-	-	53.2	-	-	49.4
	median	49.6	43.2	50.0	45.0	-	-	53.5	28.1	29.4	-	-	53.9	-	-	50.5

-: no data.

**Table 3:** Prevalence of *Vibrio* species in water samples from the North Sea, Eastern Scheldt, Wadden Sea and Veerse Meer, from 2019 to 2020 and from 2009 to 2012.

Site	Year	Number (%) of Isolates Per Species							
		*Vibrio* Total	*V. alginolyticus*	*V. parahaemolyticus*	*V. vulnificus*	*V. fluvialis*	*V. cholerae* Non-O1\non-O139	*V. mimicus*	*Vibrio* spp.
	2019		N (%)	N (%)	N (%)	N (%)	N (%)	N (%)	-
North Sea ^1^		103	91 (88)	9 (8.7)	1 (1.0)	1 (1.0)	-	-	1 (1.0)
Eastern Scheldt ^1^		108	100 (93)	7 (6.5)	-	-	1 (0.9)	-	-
Wadden Sea ^1^		105	48 (46)	52 (50)	1 (1.0)	1 (1.0)	2 (1.9)	1 (1.0)	-
	2020								
North Sea ^1^		52	48 (92)	3 (5.8)	-	1 (1.9)	-	-	-
Eastern Scheldt ^1^		66	61 (92)	3 (4.5)	-	2 (3.0)	-	-	-
Wadden Sea ^1^		58	31 (53)	18 (31)	1 (1.7)	3 (5.2)	1 (1.7)	-	4 (6.9)
Total ^1^		492	379 (77)	92 (19)	3 (0.6)	8 (1.6)	4 (0.8)	1 (0.2)	5 (1.0)
	2019								
Veerse Meer ^1,2^		43	22 (51)	21 (49)	-	-	-	-	-
Eastern Scheldt ^2^		40	36 (90)	3 (7.5)	-	1 (2.5)	-	-	-
Wadden Sea ^2^		41	26 (63)	14 (34)	1 (2.4)	-	-	-	-
	2020								
Veerse Meer ^1,2^		46	34 (74)	11 (24)	-	-	-	-	1 (2.2)
Eastern Scheldt ^2^		44	38 (86)	5 (11)	-	1 (2.3)	-	-	-
Wadden Sea ^2^		45	37 (82)	4 (8.9)	1 (2.2)	-	-	-	3 (6.7)
	2021								
Veerse Meer ^1,2^		35	16 (46)	16 (46)	-	1 (2.9)	-	-	2 (5.7)
Eastern Scheldt ^2^		25	24 (96)	-	-	1 (4.0)	-	-	-
Wadden Sea ^2^		34	22 (65)	10 (29)	1 (2.9)	1 (2.9)	-	-	-
Total ^1,2^		353	255 (72)	84 (24)	3 (0.8)	5 (1.4)	-	-	6 (1.7)
total		845	634 (75)	176 (21)	6 (0.7)	13 (1.5)	4 (0.5)	1 (0.1)	11 (1.3)
	2009–2012								
North Sea ^1^		265	172 (65)	65 (24)	5 (1.9)	2 (0.8)	8 (3.0)	-	6 (2.3)
Eastern Scheldt ^1^		310	275 (89)	26 (8.4)	-	1 (0.3)	2 (0.6)	-	6 (1.9)
Wadden Sea ^1^		173	106 (61)	41 (24)	-	8 (4.6)	12 (6.9)	-	6 (3.4)
Total ^1^		748	553 (74)	132 (18)	5 (0.7)	11 (1.5)	22 (2.9)	-	18 (2.4)

^1^ bathing site. ^2^ open sea. -: species not detected.

**Table 4:** Predicted *Vibrio* concentrations in water in different water temperature scenarios.

Water Temperature Scenario	5%	Median	Mean	95%
10 °C	0.36	61	8.9 × 10^3^	8.5 × 10^3^
18 °C	8.8	1.3 × 10^3^	1.6 × 10^5^	2.5 × 10^5^
20 °C	19	3.3 × 10^3^	3.2 × 10^5^	6.0 × 10^5^
22 °C	39	7.0 × 10^3^	8.2 × 10^5^	1.2 × 10^6^
25 °C	120	2.4 × 10^4^	2.3 × 10^6^	4.6 × 10^6^

**Table 5:** Risks of illness from water ingestion per swimming event.

Water Temperature Scenario	5%	Median	Average	95%
Child				
10 °C	9.2 × 10^−10^	4.3 × 10^−7^	7.9 × 10^−5^	1.1 × 10^−4^
18 °C	2.1 × 10^−8^	9.2 × 10^−6^	1.7 × 10^−3^	2.9 × 10^−3^
20 °C	3.8 × 10^−8^	2.3 × 10^−5^	2.8 × 10^−3^	6.7 × 10^−3^
22 °C	9.8 × 10^−8^	4.9 × 10^−5^	5.6 × 10^−3^	1.4 × 10^−2^
25 °C	2.6 × 10^−7^	1.7 × 10^−4^	1.3 × 10^−2^	5.0 × 10^−2^
Man				
10 °C	2.2 × 10^−10^	5.2 × 10^−6^	9.0 × 10^−5^	7.1 × 10^−5^
18 °C	5.5 × 10^−9^	4.9 × 10^−6^	1.2 × 10^−3^	1.8 × 10^−3^
20 °C	1.0 × 10^−8^	1.2 × 10^−5^	2.3 × 10^−3^	5.5 × 10^−3^
22 °C	2.7 × 10^−8^	2.6 × 10^−5^	4.1 × 10^−3^	9.8 × 10^−3^
25 °C	8.6 × 10^−8^	8.9 × 10^−5^	1.1 × 10^−2^	3.9 × 10^−2^
Woman				
10 °C	2.2 × 10^−10^	1.7 × 10^−7^	5.4 × 10^−5^	5.1 × 10^−5^
18 °C	5.2 × 10^−9^	3.8 × 10^−6^	9.0 × 10^−4^	1.3 × 10^−3^
20 °C	1.2 × 10^−8^	9.5 × 10^−6^	1.7 × 10^−3^	3.5 × 10^−3^
22 °C	2.8 × 10^−8^	2.0 × 10^−5^	3.2 × 10^−3^	7.1 × 10^−3^
25 °C	8.0 × 10^−8^	7.4 × 10^−5^	7.7 × 10^−3^	2.5 × 10^−2^

## Data Availability

The original contributions presented in this study are included in the article. Further inquiries can be directed to the corresponding author.

## Abbreviations

The following abbreviations are used in this manuscript:

KNMI	Royal Dutch Meteorological Institute
ASPW	Alkaline Saline Peptone Water
TCBS	Thiosulphate Citrate Bile Sucrose Agar
SNA	Saline Nutrient Agar
PCR	Polymerase Chain Reaction
MPN	Most Probable Number
QMRA	Quantitative Microbial Risk Assessment
